# Manatee: detection and quantification of small non-coding RNAs from next-generation sequencing data

**DOI:** 10.1038/s41598-020-57495-9

**Published:** 2020-01-20

**Authors:** Joanna E. Handzlik, Spyros Tastsoglou, Ioannis S. Vlachos, Artemis G. Hatzigeorgiou

**Affiliations:** 1 DIANA-Lab, Department of Electrical & Computer Engineering, University of Thessaly, Volos, 38221 Greece; 2 Department of Biology, University of North Dakota, Grand Forks, North Dakota 58202 USA; 3 Hellenic Pasteur Institute, Athens, 11521 Greece; 4 Harvard Medical School Initiative for RNA Medicine, Department of Pathology, Cancer Research Institute, Beth Israel Deaconess Medical Center, Harvard Medical School, Boston, Massachusetts 02115 USA; 5 Broad Institute of MIT and Harvard, 02142 Cambridge MA, USA; 6 Department of Computer Science and Biomedical Informatics, University of Thessaly, Lumia, 35131 Greece

**Keywords:** Computational platforms and environments, Data processing, Software

## Abstract

Small non-coding RNAs (sncRNAs) play important roles in health and disease. Next Generation Sequencing (NGS) technologies are considered as the most powerful and versatile methodologies to explore small RNA (sRNA) transcriptomes in diverse experimental and clinical studies. Small RNA-Seq (sRNA-Seq) data analysis proved to be challenging due to non-unique genomic origin, short length, and abundant post-transcriptional modifications of sRNA species. Here, we present Manatee, an algorithm for the quantification of sRNA classes and the detection of novel expressed non-coding loci. Manatee combines prior annotation of sRNAs with reliable alignment density information and extensive rescue of usually neglected multimapped reads to provide accurate transcriptome-wide sRNA expression quantification. Comparison of Manatee against state-of-the-art implementations using real and simulated data demonstrates its high accuracy across diverse sRNA classes. Manatee also goes beyond common pipelines by identifying and quantifying expression from unannotated loci and microRNA isoforms (isomiRs). It is user-friendly, can be easily incorporated in pipelines, and provides a simplified output suitable for direct usage in downstream analyses and functional studies.

## Introduction

The discovery of short functional RNA classes such as microRNAs (miRNAs) and small interfering RNAs (siRNAs) revealed their involvement in pervasive regulation of gene expression and inaugurated the RNA revolution. NGS techniques offer a powerful high-throughput means for the quantification and discovery of many sRNA classes^1^. sRNA-Seq has been established as the gold standard technique for high-throughput detection and quantification of sRNAs typically ranging between 18 and 35 nucleotides in length, enabling expression studies of sRNA species, as well as for the discovery of novel sncRNAs. miRNAs have been the focal point of such analyses, since they play a pivotal role in post-transcriptional regulation of gene expression^2^ controlling pathways in health and disease^3,4^. Other sRNAs identified in NGS experiments, such as ribosomal RNAs (rRNAs), transfer RNAs (tRNAs) and small nucleolar RNAs (snoRNAs), were usually conceived as findings of secondary significance. However, recent studies have provided insight into novel biological roles of such sRNAs^5–7^. Using relevant approaches, new sRNA families with biological functions that are still under debate have been discovered. tRNA-derived RNA fragments (tRFs), a novel class of sRNAs second in abundance only to miRNAs^5^ or box C/D snoRNAs^7^ comprise characteristic examples of such classes. The majority of tRF sequences are derived from precise cleavage and processing at the 5′ or 3′ end of mature or precursor tRNAs, and studies indicate their possible involvement in miRNA-like RNA targeting as well as global translational suppression^8^. snoRNAs, known to serve functions in RNA modification processes^9^, have been recently shown to host specific miRNA-like short RNAs and have been found deregulated in various diseases and malignancies^6,7^. Hence, accurate quantification and analysis of the full sRNA spectrum is of great interest.

Small RNA-seq data contain a plethora of processing and maturation products potentially including yet unknown RNA species^10^. The non-coding RNA (ncRNA) field is rapidly expanding with an increasing number of newly identified and biologically relevant and important ncRNAs^11^. These considerations highlight the need for sensitive, accurate and efficient bioinformatics tools that can properly handle any kind of small ncRNA present in sRNA-seq datasets.

Currently the analysis of sRNA-Seq data is not as mature as for longer RNAs, and their usefulness is impacted by major hindrances. Particularly, the short length (usually ~18–30 nt) of sRNA-Seq reads introduces the problem of multi-mapping, where a single read may align to multiple genomic locations with equal alignment scores. This issue is exacerbated if we consider that many sRNAs are transcribed from repeat loci^12^. As a consequence, the most common approaches adopted for RNA-Seq data^13^ cannot be successfully applied here: retaining only uniquely aligned mappings^14^ leads to the omission of a significant portion of reads, while other strategies such as equal distribution^15,16^, random read placement^17^ or reporting all possible alignment positions of multimapping reads^18^, inevitably leads to incorrect or indirectly quantifiable results^13^. Additionally, the analysis of numerous intermediate and terminal products of sRNA biogenesis, as well the potential discovery of yet unknown RNA species in sRNA-Seq data, remains undermined with current approaches^11^.

State-of-the-art methods employ direct alignment against known miRNA or sRNA annotations and not on the genome, in order to diminish the extent of multi-mapping^13^. However, these methods are bound to quantifying only known sRNAs, while reads that could align better in other genomic loci are forced to map with lower scores in the reduced search space^19^. The ambiguity of the genomic origin of sRNAs may also lead to cross-mappings, in which a short RNA originating from one locus is partially or completely assigned to a different location^20^. Moreover, most available algorithms are dedicated to studying a single sRNA biotype^21^, which further restricts the alignment space and can lead to the misclassification of reads.

Current implementations for sRNA-Seq quantification can be divided in two categories based on their analysis scope: those that quantify only a single sRNA family, such as miRDeep2^21^, and those pursuing to cover the broad sRNA space such as miRge^22^, sRNAbench^23^, and ShortStack^24^. miRDeep2 is an extensively used tool dedicated entirely to miRNA quantification, while miRge prioritizes the miRNA biotype over the rest of the sRNAs by utilizing a step-wise alignment strategy against mature miRNAs, miRNA hairpins, ncRNAs, mRNAs, and a modified miRNA library. Implementations such as sRNAbench or ShortStack have sought to address the positioning of multimapping reads in a more refined manner. sRNAbench either assigns multimaps wholly to all their mapping positions, or divides their counts equally between them. Both practices could potentially lead to misinterpretation of transcript expression, especially in cases where multimapping positions pertain to different RNA biotypes. Read mapping, as implemented in the ShortStack tool, is based on local-weighting read alignments. The attempt for improved multimaps placement in ShortStack, relies on unique or fractional weighting schemes and their calculated probabilities for each alignment. This probabilistic placement of multimaps may lead to disparate expression profiles in repeated executions of the same sample. Additionally, highly multi-mapped reads, which can still carry biologically important information, are discarded by this approach.

Since multimaps are a major obstacle for accurate analysis of sRNA-Seq datasets, we first analyzed 30 sRNA-Seq libraries from diverse tissues to assess the distribution of uniquely aligned and multimapping reads across samples. We further examined the mappings with respect to existing annotation and identified interesting aspects of sRNA-Seq data and assessed the underlying complexity in the placement of multimapping reads. Based on our findings, we implemented the sMAll rNa dATa analysis pipElinE (MANATEE) for detection and quantification of known and unknown small RNAs by efficiently rescuing and utilizing multimapping reads. Manatee is not limited to a single sRNA class and achieves highly accurate results, even for elements residing in heavily repeated loci, by making balanced use of existing sRNA annotation and observed read density information during multi-mapping read placement. Manatee does not prioritize any particular sRNA type, enabling the accurate quantification of diverse RNA classes. Additionally, Manatee exploits sRNA-Seq reads to detect expressed unannotated genomic loci that could harbor still unknown sRNA products. The user-friendly pipeline of Manatee returns ncRNA expression counts that can be directly utilized in downstream analyses, such as differential expression analyses, rendering it easily integrable in larger bioinformatics workflows.

## Results

### Multimaps analysis

**Figure 1:**
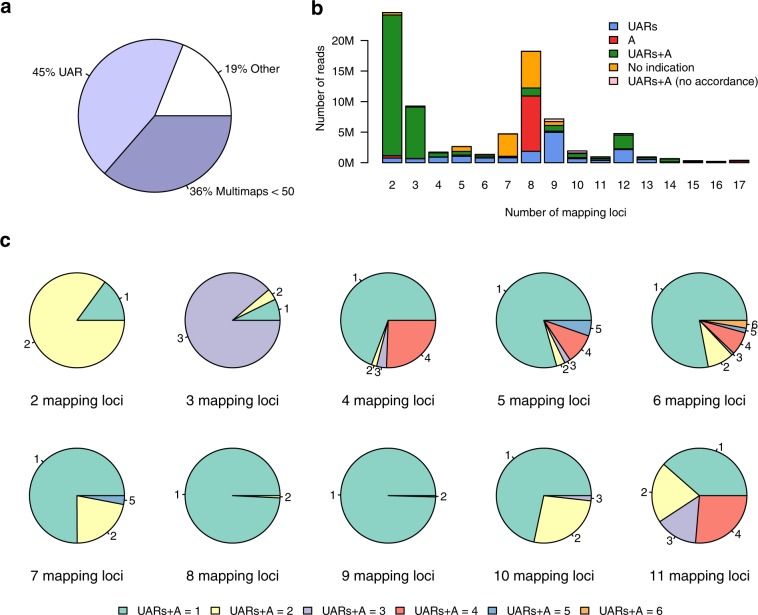
Frequency, proportions, and characteristics of multimaps in sRNA-Seq libraries. (**a**) The average number of UARs, multimaps, and other reads (i.e. unaligned/multimaps exceeding the defined threshold) across all samples. (**b**) Multimap read categories based on available annotation and UARs. Colors mark five examined cases where each multimap is screened for available annotation and UARs. (**c**) Proportion of multimaps and the number of their mapping regions with both UAR clusters and available annotation.

In order to study the characteristics of multi-mapping reads, we performed an initial analysis of 30 distinct human sRNA-Seq libraries derived from hepatoblastoma, liver, brain, gallbladder, colon, lung, pancreas, skin, tongue, thyroid, and heart tissue, embryonic stem cells, as well as MCF7 and HepG2 cell lines, in order to assess the extent of multimaps and uniquely aligned reads (UARs) in sRNA-Seq datasets (Supplementary Table 1). All the above libraries were obtained from Gene Expression Omnibus^25^ (GEO). Figure 1a presents the average percentage of UARs, multimaps, and unaligned reads across the samples. Five examined cases of positioning multimaps were based on reads with 2 to 17 multimapping regions (Fig. 1b). According to the analyzed cases, a multimap may fall into:

1. unannotated regions of UAR clusters (denoted as blue in Fig. 1b)
2. annotated regions lacking UAR clusters (red)
3. annotated regions that also contain UAR clusters (green)
4. unannotated regions that also lack UAR clusters (orange)
5. annotated regions and regions with UAR clusters with no concordance (pink).

Case 3, which includes multimaps falling into regions with both existing annotation and UAR clusters, was further analyzed and examined for the number of such regions per multimap (Fig. 1c). For example, the majority of multimaps with two possible mapping loci had UARs and annotation for both mapping positions. The majority of reads with four possible mapping loci had UARs and annotation for one of the four mapping positions. The distribution of the five examined cases across numbers of multi-mapping regions was evaluated across different numbers of randomly selected samples to estimate whether the selected sample size introduced biases in the analysis of multimaps in sRNA-Seq libraries (Supplementary Fig. 1). As shown in Supplementary Fig. 1, the distributions of the multimap cases across different sample sizes are consistent, providing confidence that the selected sample size is sufficient in the current analysis of small reads.

A large portion of sRNA-Seq reads (36%) in the analyzed datasets mapped to multiple genomic loci (Fig. 1a). 19.7% of total multimaps fell into regions with UARs lacking annotation and for 15.2% no straightforward information of positioning or annotation was available (Fig. 1b). Algorithms based on genomic alignment that rely entirely on UAR information, may fail to account for cases of multimaps that could otherwise be assigned to existing annotation (red in Fig. 1b, 13.3% of total multimaps). On the other hand, multimaps assigned to more than one genomic feature using annotation from a broader spectrum of ncRNAs (Fig. 1c) showed that tools dedicated entirely to a specific RNA biotype may be biased towards that type (Supplementary Fig. 2).

### Manatee

**Figure 2:**
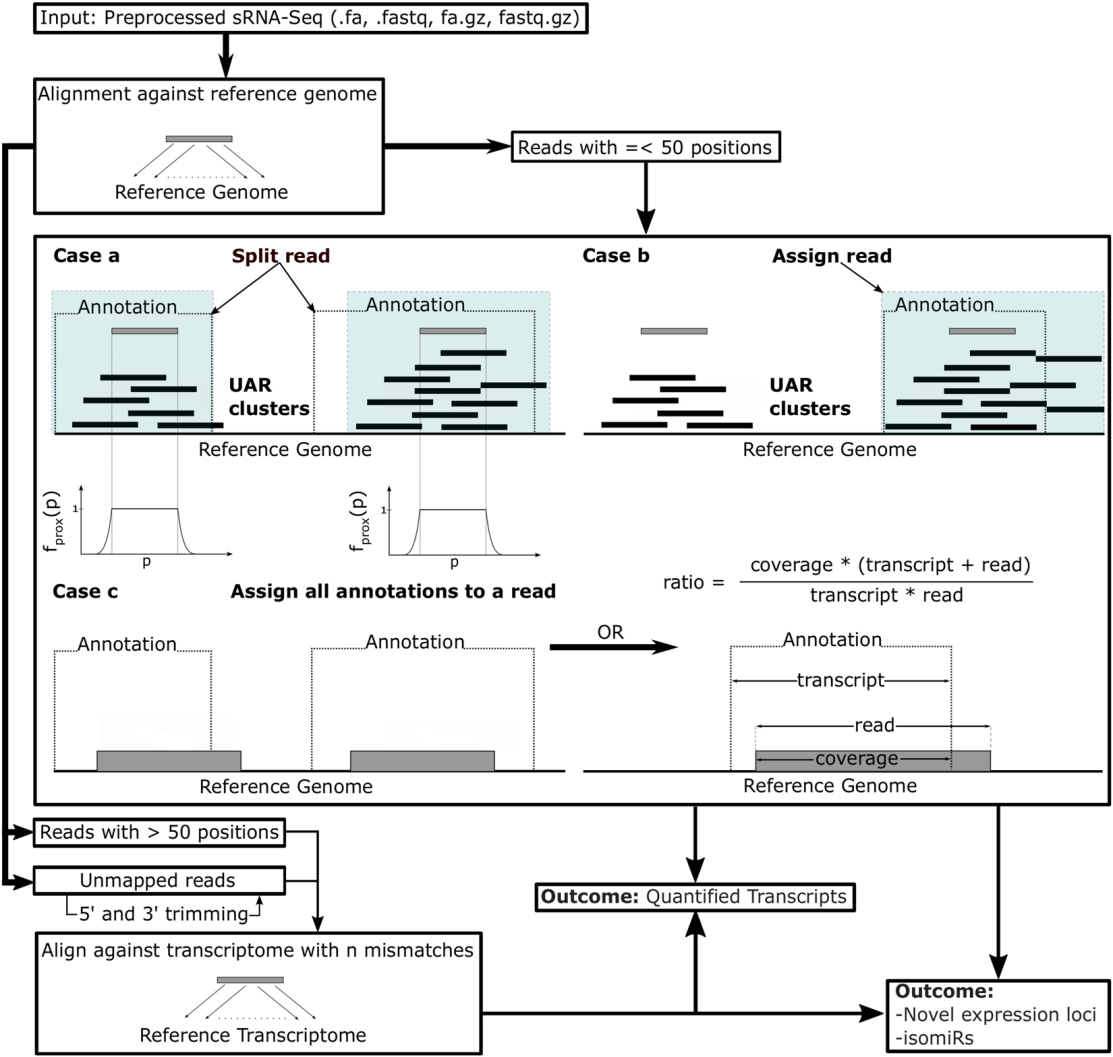
Manatee workflow. Reads with up to 50 multi-mapping positions are either: (**a**) split among their annotated and UAR-containing loci according to Eq. 1, (**b**) assigned to regions containing both annotation and UARs, or (**c**) assigned to loci with existing annotation. In case of (**c**), if an annotated miRNA is within the annotated loci, a ratio for selecting the best fitted transcript is used to prioritize mature miRNAs over precursors. Reads with more than 50 mapping positions, reads which could not be mapped to the genome, and reads that could not be assigned to regions with existing annotation and UARs are aligned against the transcriptome with gradual increment of allowed mismatches. The output results contain quantified transcripts, putative novel expression loci, and isomiR sequences.

The conclusions yielded by the multimaps analysis constituted the basis for the Manatee algorithm which attempts to approach the multimap issue by simultaneously incorporating information from UARs and existing annotation. We aimed to combine into a single step the crucial information of uniquely mapped reads and annotation without prioritizing any particular sRNA type (Fig. 2 and Methods section). The algorithm also attempts to salvage highly multimapping and unaligned reads, which are usually discarded in many available sRNA analysis pipelines. Additionally, Manatee exploits sRNA-Seq reads to detect expressed unannotated genomic loci that could harbor yet unknown small RNA products.

### Comparison to other methods using simulated data

The accuracy of Manatee was initially evaluated using a simulated short read dataset (https://github.com/jehandzlik/Manatee/tree/simulatedData). Bowtie v1^17^ was used as a baseline, since it is a commonly used aligner in sRNA-Seq pipelines, while miRge, ShortStack, and sRNAbench were employed as state-of-the-art approaches in the evaluation. miRge and ShortStack perform read alignment with singular functionality, against sRNA annotation (miRge) and against the genome (ShortStack), while sRNAbench extends the functionality of miRanalyzer by applying genomic/transcriptomic alignment of multiple sRNA types in a hierarchical step-wise manner. Those diverse approaches of sRNA quantification constitute attractive candidates for direct comparisons with the Manatee algorithm. miRge, ShortStack, sRNAbench (genomic alignment mode), and Manatee were executed under their default settings. Bowtie was executed permitting a maximum of 1 mismatch and up to 5 multimaps, while transcript quantification was performed with HTSeq-Count^14^ using the intersection-nonempty mode and “nonunique all” parameter. The selected parameters for both Bowtie and HTSeq-Count were found to be optimal for the input in question.

**Figure 3:**
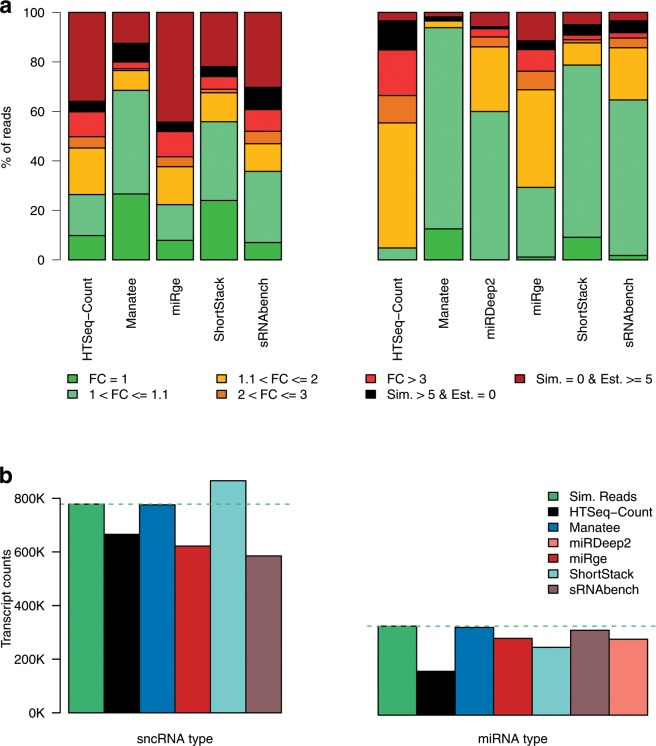
Tool evaluation statistics for simulated sRNA-Seq data. (**a**) Fold changes for simulated vs. estimated transcript counts for evaluated tools utilizing all small ncRNA species or only miRNAs. Fold change of 1 denotes no difference between the simulated and the calculated counts. Sim. > 5 & Est. = 0 denotes percentage of reads where the simulated transcript counts >5 were estimated as zeros by the examined tools. Sim. = 0 & Est. > = 5 relates with proportion of estimated transcript counts >5 for which the true simulated count was zero. (**b**) Comparison between the ground truth count sum of simulated reads and the total estimated transcript counts across implementations.

Estimated sRNA counts for HTSeq-Count, Manatee, miRge, ShortStack, and sRNAbench were contrasted to the ground truth (i.e. simulated counts) (Fig. 3a). All tools tend to over-estimate numerous transcripts that have zero abundance in the simulated dataset (Fig. 3a, Sim. = 0 & Est. > = 5). However, the opposite behavior was observed at the other end of the spectrum: expressed and highly expressed transcripts were not assigned any reads (Fig. 3a, Sim. >5 & Est. = 0). Among the tested tools, counts estimated by Manatee appeared closest to the simulated abundances (Fig. 3).

Manatee is not only able to map and accurately quantify diverse sRNA classes, but it also fares favorably when compared to methods specifically designed for miRNAs. miRDeep2, which uses Bowtie to map sequencing reads against precursors and discards or assigns multimaps equally to their valid loci, was executed against the same dataset, with default settings. These results vividly depict that Manatee users can quantify and investigate underexplored small RNA classes, while also obtaining accurate and robust results for miRNAs (the superstar of sncRNA class).

The sum of simulated transcript counts was contrasted against the estimated counts by the six tools. ShortStack displayed tendency for count inflation, while HTSeq-Count, miRge, miRDeep2, and sRNAbench underestimated transcript counts (Fig. 3b). Precision metrics were also calculated to assess the performance of the examined algorithms by comparing simulated to estimated read counts for the entire pool of small ncRNA transcripts, as well as for miRNAs only (Table 1). Root-mean-squared deviation (RMSD), distance metrics, and correlation coefficient values computed for estimated counts versus the ground truth all indicate that Manatee outperforms the other implementations by providing less inflated/deflated transcript counts that are more closely associated with the simulated counts. A major driving force for this increase in accuracy is the rescue of multimapping reads. Manatee aligns against the genome using Bowtie but rescues efficiently the multimapping reads by assigning them to the most probable loci. In comparison, the use of uniquely aligned reads from Bowtie, a commonly used approach, is one of the lowest performers.

**Table 1:** Performance metrics for the accuracy of evaluated implementations using simulated data.

Tool	RNA type	RMSD	Jaccard distance	Euclidean distance	Pearson correlation	Spearman correlation
HTSeq-Count	small ncRNAs	372.614	0.298	20439.478	0.798	0.577
Manatee		**341.494**	**0.173**	**15730.981**	**0.879**	**0.796**
miRge		408.08	0.503	26553.417	0.641	0.581
ShortStack		456.499	0.271	20939.313	0.801	0.655
sRNAbench		361.399	0.395	22805.388	0.744	0.556
HTSeq-Count	miRNAs	369.164	0.442	8813.660	0.529	0.392
Manatee		**107**	**0.031**	**2556.831**	**0.929**	**0.954**
miRge		249.67	0.216	6419.008	0.731	0.684
ShortStack		236.657	0.151	5738.626	0.683	0.737
sRNAbench		215.574	0.138	5218.504	0.752	0.725
miRDeep2		155.313	0.078	3867.274	0.893	0.827

### Comparison to other methods using real sRNA-Seq data

**Figure 4:**
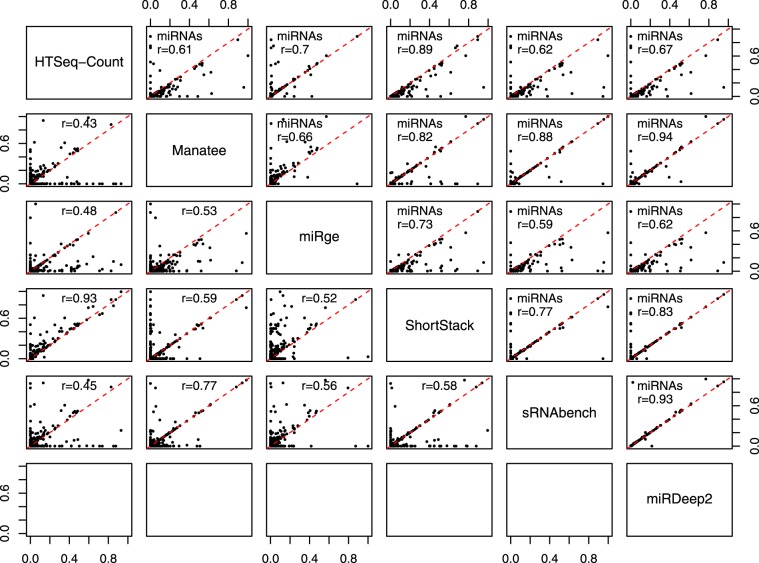
The analyzed sRNA-Seq sample was compared across 5 methods for all sRNA types (lower left panels) and across 6 methods for miRNAs (upper right panels). Pearson correlation was calculated for each pair of compared tools and denoted on each plot with the red line indicating the perfect correlation.

Although using simulated datasets offers the advantage of knowing the true transcript abundance, this practice can be prone to shortcomings (e.g. lack of complexity observed in real data sets). The alternative of using real data, allows the examination of agreement among different quantification algorithms. For this reason, we used sRNA-Seq data derived from breast cancer MCF7 cells (Study ID: SRP060224, Sample ID: SRR2084358) and obtained from GEO to cross-correlate the compared sRNA/miRNA quantification methods using Pearson correlation (Fig. 4). Seven genomic features exhibiting read counts above 10,000 reads in all executions were removed from the comparison as outliers (Supplementary Table 3). Removing these few features excluded factors that would have spuriously inflated and skewed the correlations between the estimated transcript counts among the examined tools.

Real data enabled the comparison and the assessment of concordance between the tools. In the total sRNA space, the highest concordance (r = 0.93) was observed for the performance of Bowtie + HTSeq-Count and ShortStack, followed by the Manatee-sRNAbench pair-wise comparison (r = 0.77). For miRNAs, Manatee exhibited >0.8 correlation coefficient with ShortStack, sRNAbench and miRDeep2, and exhibited the highest correlation (r = 0.94) with miRDeep2 which is the reference tool in miRNA quantification. When comparing the total sRNA transcriptome results, a substantial divergence between estimated counts was observed across executions. These findings indicate that the tools may each have intrinsic properties that result, at least in some cases, in misclassification and erroneous quantification of sRNAs.

### Unannotated clusters

**Figure 5:**
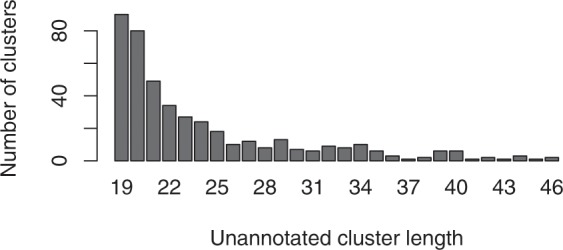
Length distribution of uniquely aligned read clusters lacking annotation in real sRNA-Seq sample.

Manatee supports the detection of expressed unannotated loci that can be used to identify novel sRNAs and sRNA classes in diverse research settings. Execution of Manatee with default settings on the MCF7 sRNA-Seq sample (Study ID: SRP060224, Sample ID: SRR2084358) detected a total of 588 unannotated clusters. 503 clusters with cluster length <50 nt are shown in Fig. 5 (mean reads per cluster *µ* = 35.06 and *σ*^2^ = 114.05). Users aiming to proceed with the detection of novel sRNA genomic loci are strongly advised to first overlap the detected clusters with protein-coding exon annotation in order to exclude putative products of mRNA degradation events^26^. Following this filtering step and using coding annotation derived from Ensembl v85^27^, 74 clusters remained as highly promising loci for further investigation.

## Discussion

Small RNA-Seq experimental datasets require extra caution during the alignment and quantification processes compared to RNA-Seq libraries due to technical obstacles arising from small read and transcript lengths. Short sequences tend to map to more than one genomic region, thus affecting transcript quantification. Furthermore, numerous small RNAs (miRNAs, tRNAs, rRNAs, etc.) can originate from repeat loci. Variations and post-transcriptional modifications introduce an additional layer of complexity to the detection of proper mapping loci. Many available tools for sRNA-Seq analysis focus on specific short RNA types, at the expense of other subclasses and the breadth of the investigation. Alignments solely against transcripts neglect expression patterns which could bear information about their origin. Alternatively, alignments against the genome are hindered by the lack of unique assignment of numerous sequenced reads. The extensive analysis of small RNA alignments in 30 sRNA-Seq samples revealed that a substantial proportion of sRNA-Seq reads are indeed multimapping. Further analysis showed that many of these positions may refer to different RNA classes, supporting the idea that placement of short reads should include diverse RNA biotypes. Manatee is an algorithm for quantification of sRNA-Seq data based on a novel way of multimaps rescue based on a step-wise approach, exploiting (i) available annotation and (ii) reliable/robust density information towards an optimized multi-mapping read placement which as shown here, improves the accuracy of small RNA quantification. Compared against standard and state-of-the-art methods, Manatee seems to outperform all tested methods even those that are specific to a single sRNA class (e.g. miRNA-specific methods). Furthermore, it enables the detection and quantification of putative expressed small RNA loci lacking annotation. Future expansions of the algorithm could include the incorporation of tolerance against common post-transcriptional modifications or indels to further boost the precision of transcript quantification in a broader and more realistic alignment space. Manatee provides an improved approach to quantify transcripts present in sRNA-Seq data by combining reliable information inferred from UARs and transcript annotation, to more accurately guide the placement of multi-mapping reads. It is an efficient and user-friendly tool that can be a significant aid in small RNA studies.

## Methods

### Multimap analysis

Quality-check and pre-processing of all libraries utilized in multimaps analysis was performed as in Vlachos *et al*.^28^. In brief, dataset quality control was performed using FastQC^29^. Cutadapt^30^ was used for adapter and contaminant removal. Reads were mapped against the GRCh38 human reference assembly using Bowtie. UARs and multimaps with up to 50 genomic positions were retained for further analysis. Clusters of UARs were created across the genome for each sample. UARs were considered as reads mapping uniquely to the genome with one allowed mismatch and Bowtie “best strata”^17^. Genomic position of UAR includes the information about the mapping chromosome, strand, start, and end position. The minimum density of a UAR cluster was set to one read. Non-coding annotation available in Ensembl v85^27^, GtRNAdb 2.0^31^, and miRBase v21^32^ was used to construct a reference for genomic features. Specifically, long ncRNA (lincRNA), mitochondrially encoded rRNA (mt-rRNA), mitochondrially encoded tRNA (mt-tRNA), processed transcript, rRNA, small cytoplasmic RNA (scRNA), snoRNA, small nuclear RNA (snRNA), and vault RNA (vtRNA) gene types were derived from Ensembl, tRNAs were derived from GtRNAdb, while miRNA precursor and mature annotation was derived from miRBase. A minimum 1 nucleotide overlap between the genomic position of aligned read and an annotated transcript was required to assign the read to that specific transcript. All transcripts and UARs were extended by 50nt at each end to allow flexibility in the assignment of reads without adding bias.

### Manatee algorithm

#### Input

Manatee requires FASTQ/FASTA sRNA-Seq data files that have been pre-processed for 3′ adapter and barcode removal. Genomic annotation for ncRNAs is required as input in GTF format with the following tags in the attributes field: gene_name, gene_id, and gene_biotype.

#### Alignment and quantification

The full outline of NGS reads abundance estimation adopted by Manatee is provided in Fig. 2. Mapping of sequencing reads is carried out using Bowtie aligner. In the primary phase, reads aligned uniquely to the genome are used to form the UAR clusters across the genome. Multimaps are assigned to loci based on the following approach:

1 $${f}_{split}({x}_{i},{y}_{i})=\frac{{f}_{score}({x}_{i},{y}_{i})}{{\sum }_{i=1}^{MML}{f}_{score}({x}_{i},{y}_{i})}$$

2 $$\hspace{16pt}{f}_{score}({x}_{i},{y}_{i})=\mathop{\sum }\limits_{p={x}_{i}-r}^{{y}_{i}+r}{f}_{cov}(p)\cdot {f}_{prox}(p)$$

3 $$\hspace{49pt}{f}_{prox}(p)=\{\begin{array}{c}1,{x}_{i}\le p\le {y}_{i}\\ \frac{1}{{e}^{({x}_{i}-p)/n}},{x}_{i}-r < p < {x}_{i}\\ \frac{1}{{e}^{(p-{y}_{i})/n}},{y}_{i} < p < {y}_{i}+r\end{array}\,$$

where *x*_*i*_ and *y*_*i*_ are the start and end placement positions of the multimap *i* and *r* is the range in the close proximity of the read (default 50). Function *f*_*cov*_ denotes the UAR density at genomic position *p* and *f*_*prox*_ assigns weights to *f*_*cov*_ based on the position *p* within the genomic region [*x*_*i*_ − *r*, *y*_*i*_ + *r*]. The multimap is split across its valid multi-mapped loci (*MML)* according to the score calculated using function *f*_*split*_. *n* denotes the relevance of approximate density distribution and is set by default to 10. For multimaps with non-matching annotation and positioning of UAR clusters, annotation is preferred and used to guide the final placement of the reads. If a multimap falls into regions which are annotated completely or partially, all relevant transcripts are noted in the output file in the form of alternative transcripts. In case where at least one annotated miRNA is present among those features, the read is assigned to the transcript which exhibits the highest coverage score (ratio):

4 $$ratio=\frac{coverage\cdot (transcript\,length+read\,length)}{transcript\,length\cdot read\,length}$$

Coverage is the number of overlapping nucleotides between the annotated feature (transcript) and the read length. The ratio heuristic prioritizes the annotation with the highest coverage, while also considering read and transcript lengths.

#### Salvaging reads by secondary transcriptome alignment

Reads that exceed the multi-mapping threshold and reads that could not be mapped to the genome are additionally aligned against the transcriptome based on the provided annotation. In the latter case, the number of allowed mismatches is gradually augmented (maximum default 3). In both cases, reads that can be assigned to transcripts with existing mapping densities calculated in previous steps are assigned to those transcripts. If no expression estimates exist, up to five transcripts with the highest mapping quality are retained and assigned as alternative transcripts.

#### IsomiR detection

All reads assigned and quantified as miRNA type are retained and stored in a separate output file. Each detected putative isomiR sequence is stored independently along with its estimated count. Since each read represents the actual sequence of the sRNA molecule, it can be used to identify diverse miRNA modifications, such as post transcriptional modifications, 5′ and 3′ templated additions, or single nucleotide variations. Manatee saves all reads and clusters them per miRNA. These results may serve as the foundation for a downstream isomiR analysis.

#### Detection of novel unannotated expression loci

UARs mapping to loci lacking genomic features are organized into read clusters based on their genomic positions. Manatee identifies clusters as genomic regions which contain at least five reads and no gap longer than 50 nt between consecutive reads with the default parameters, which can be altered by the user. The output of this step is a single file comprising the unannotated genomic loci and their associated read counts.

#### Output

Manatee execution generates three, tab-separated count files (Transcripts, IsomiRs, Unannotated Loci) that can be readily incorporated in downstream analysis pipelines, such as counts-based methods for differential expression analyses (e.g. limma^33^, DESeq 2^34^, or edgeR^35^). Apart from information regarding the quantified transcripts (“Transcript ID”, “Biotype”, “Transcript Name”) and estimated non-normalized counts (“Count” column), the files provide also other useful metrics, such as reads per million reads mapped on small RNAs (“RPM”) and uniquely mapped reads (“Unique Reads”). If the reads could have been assigned to a different transcript with equal probability, its ID is provided in the “Alternative Transcripts” column.

Similarly, the isomiR results file provides detailed information about the identified expressed isomiRs and comprises the “Transcript Name”, “Count”, and “RPM” columns. The isomiR-specific “Sequence” column describes the genomic sequence of reads assigned to the identified isomiR.

The third output file describes in detail novel expressed unannotated loci and comprises the following columns: “Chromosome”, “Strand”, “Start”, “End”, “Cluster Length”, and “Count”. The columns “Chromosome”, “Strand”, “Start”, and “End” provide genomic location information of the unannotated clusters. “Cluster Length” is the nucleotide length of the unannotated cluster and “Count” is the number of reads that were assigned to the unannotated feature.

### Simulated data

A simulated short read dataset (https://github.com/jehandzlik/Manatee/tree/simulatedData) was created using random sampling with a Monte Carlo inversion technique. Human annotation was derived from Ensembl v85, GtRNAdb 2.0, and miRBase v21. Three randomly selected sRNA-Seq libraries (Supplementary Table 2) obtained from GEO were also employed in the process. Samples were aligned against GRCh38 human reference assembly after 3′-adapter sequences were removed using Cutadapt. Since processed sRNA fragments/features are derived from their precursors by biogenesis/cleavage mechanisms that are distinct to each biotype, simulated reads were designed to follow this rationale. Based on uniquely aligned reads observed in the real data, probability mass functions (PMFs) were created for each biotype describing the read start positions. Nine different PMFs were created for the following RNA types: miRNA, tRNA, mt-tRNA, rRNA, mt-rRNA, snRNA, snoRNA, lincRNA and processed transcript. Likewise, SNPs and read lengths for each sRNA type were also estimated based on PMFs of UARs. More details on the creation process and the dataset characteristics are available in the Supplementary File (Section “Simulated Reads Analysis” and Supplementary Figs. 3–5).

## Supplementary information

Supplementary File.

## Data Availability

The simulated data set is available at https://github.com/jehandzlik/Manatee/tree/simulatedData.

## Code availability

Manatee code is available at the project home page: https://github.com/jehandzlik/Manatee.
