# Supplementary material for: Manatee: detection and quantification of small non-coding RNAs from next-generation sequencing data
Source: Sci Rep. 2020 Jan 20;10:705. doi: 10.1038/s41598-020-57495-9 (PMC6971259; doi:10.1038/s41598-020-57495-9)
Supplement: Supplementary file 1 — Supplementary File. [file 41598_2020_57495_MOESM1_ESM.pdf]

Manatee: detection and quantification of small non-coding RNAs from next-generation sequencing data

Joanna E Handzlik[1,2], Spyros Tastsoglou[1,3], Ioannis S Vlachos[4], and Artemis G Hatzigeorgiou[1,3,*]

[1]DIANA-Lab, Department of Electrical & Computer Engineering, University of Thessaly, Volos 38221, Greece

[2]Department of Biology, University of North Dakota, Grand Forks, North Dakota 58202, U.S.A.

[3]Hellenic Pasteur Institute, Athens 11521, Greece

[4]Harvard Medical School Initiative for RNA Medicine, Department of Pathology, Cancer Research Institute, Beth Israel Deaconess Medical Center, Harvard Medical School, Boston, Massachusetts 02115, U.S.A.

* Corresponding author: arhatzig@inf.uth.gr

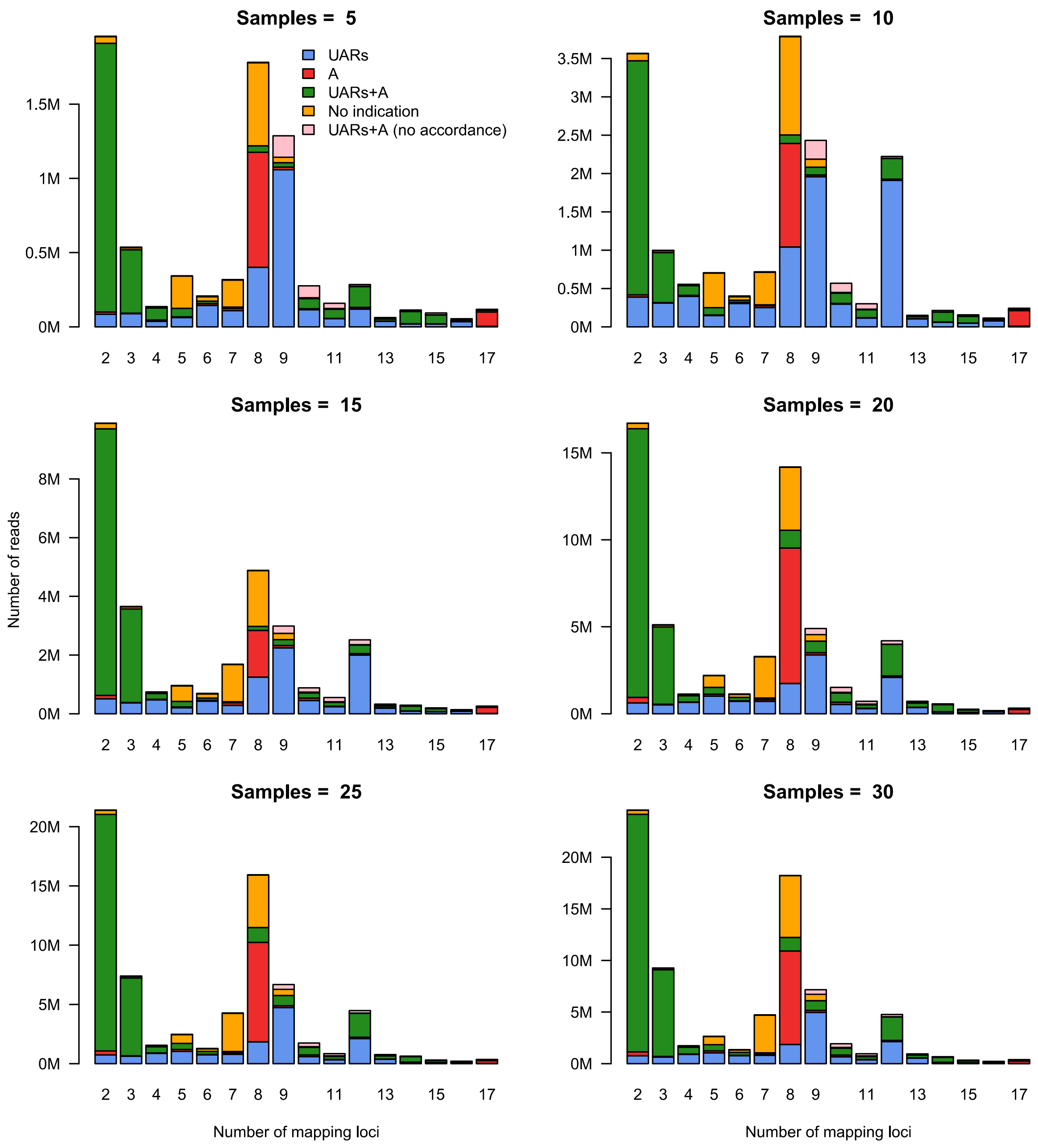

Supplementary Figure 1. Multimap read categories based on available annotation and UARs as estimated for different number (5, 10, 15, 20, 25, and 30) of randomly selected sRNA-Seq libraries. Colors mark five examined cases where each multimap is screened for available annotation and UARs.

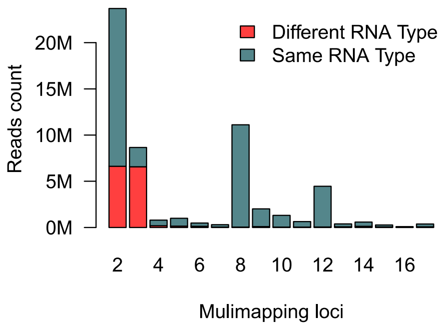

Supplementary Figure 2. Concordance between multimapping loci and corresponding biotypes. Proportion of multimapping reads with associated annotation of the same or different RNA biotype for each of the multimapping loci. A substantial number of reads with two and three multimapping positions associate with annotations of dissimilar small RNA types.

Simulated Reads Analysis

Simulation of sRNA-Seq reads was based on parameters and statistics estimated from three randomly selected sRNA-Seq libraries (Supplementary Table 2). The proportion of sRNA biotypes present in the simulated dataset was based on predefined probabilities (Supplementary Fig. 3a). The start position of reads for each genomic feature was sampled from cumulative distribution functions (CDFs) created for each sRNA biotype separately and based on the positioning of UARs in the dataset utilized in the simulation (Supplementary Fig. 4). To determine the uniquely aligned clusters of reads, the input data was pre-processed as in Vlachos *et al.*[1] and aligned against the GRCh38 genome assembly using Bowtie v1[2] with 1 allowed mismatch. Reads presenting ≥ 20% overlap with a specific genomic feature were assigned to that feature and used in the CDF estimation. For example, according to the generated miRNA CDF, simulated reads for this biotype have a high probability (87%) to start at position 0 of the mature miRNA annotation. The probability of a simulated read to bear a sequence modification was based on the observed 3' and 5' modifications (non-templated additions) and Single Nucleotide Polymorphisms (SNPs) in UARs in the examined sRNA-Seq libraries (Supplementary Fig. 3b). Similar to the case of the read starting positions, SNP position and variation, non-template additions and read lengths were also sampled from generated CDFs based on the respective information retrieved from UARs (Supplementary Fig. 5). Supplementary Figure 5a-b presents the CDFs of SNP positions and transcript lengths excluding the lengths of miRNA type, Supplementary Fig. 5c-d displays the probabilities of non-template modification types, while Supplementary Fig. 5e shows the probability of SNP variation in case a simulated read has SNP. Integer lengths of miRNA transcripts were sampled from a truncated normal distribution with $\mu = 22$, $\sigma^2 = 1$, $a = 18$, and $b = 24$, where (a, b) is the distribution interval. Read counts generated for each transcript were sampled from negative binomial distribution corresponding to selected for simulation transcript biotype. Count of the simulated read X_{ij} for transcript i and biotype j was sampled from

$$X_{ij} \sim Negative\ Binomial\ (mean = \mu_{ij}, variance = \mu_{ij} + \frac{1}{r_{ij}}\mu_{ij}^2)$$

where $\frac{1}{r_{ij}}$ is a dispersion parameter which was set to $r_{ij} = \frac{\mu_{ij}}{4}$ by default for every RNA biotype used in the simulation. Mean μ_{ij} of transcript i and biotype j was estimated based on the UARs present in the input libraries which mapped to selected for simulation biotypes.

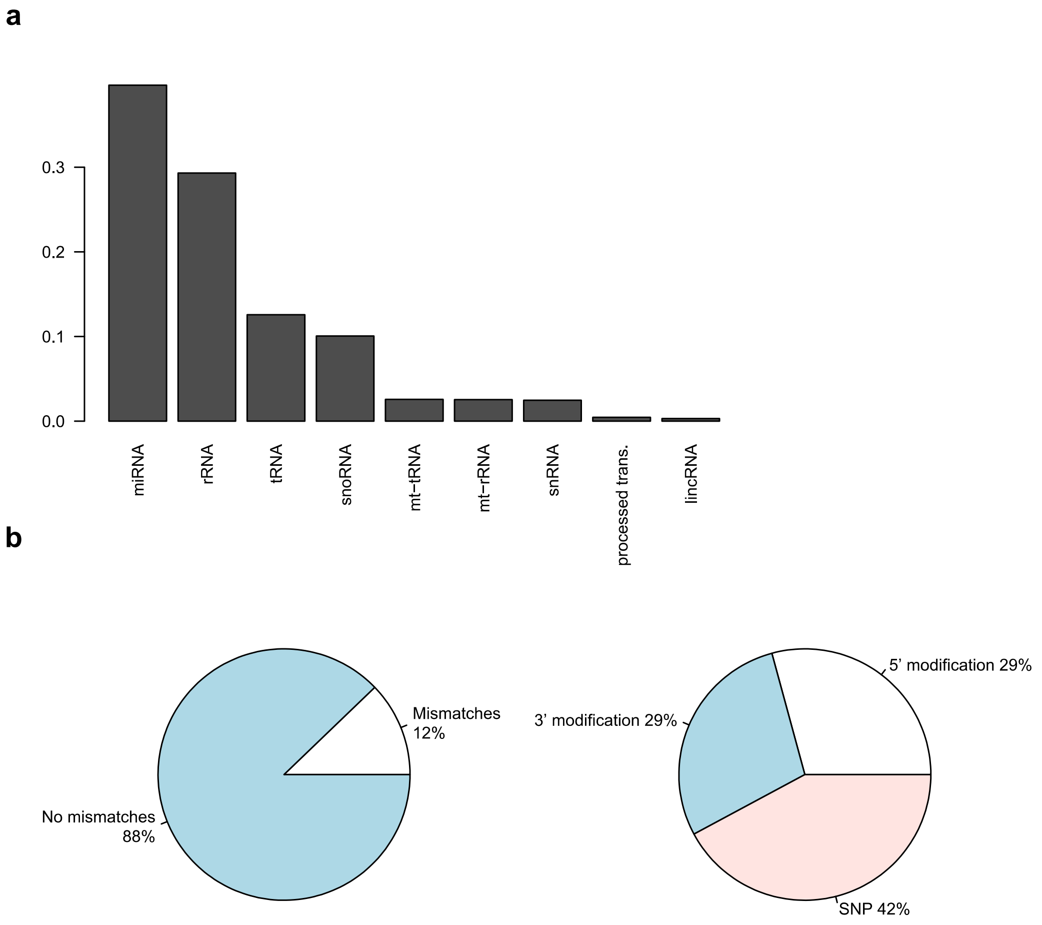

Supplementary Figure 3. sRNA biotype distribution present in the simulated data and statistics of sequence modifications in the selected sRNA-Seq libraries. (a) Proportion of sRNA biotypes present in the simulated dataset. (b) Proportion of reads with mismatches, as well as SNPs, 3', and 5' non-templated additions observed across the sRNA-Seq libraries used in the simulation process.

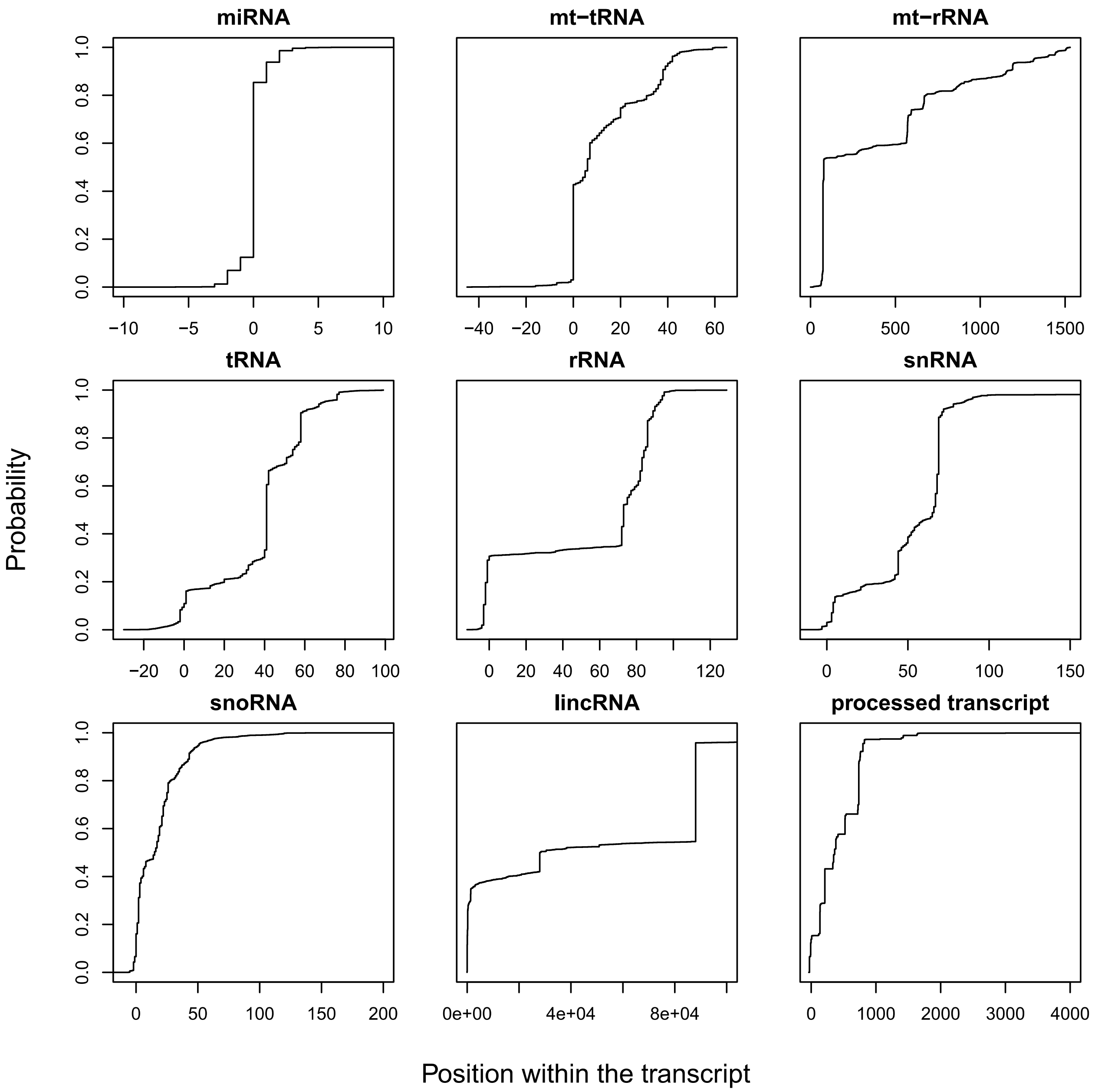

Supplementary Figure 4. CDFs of starting position of reads per biotype, based on UARs present in the sRNA-Seq libraries selected for simulation.

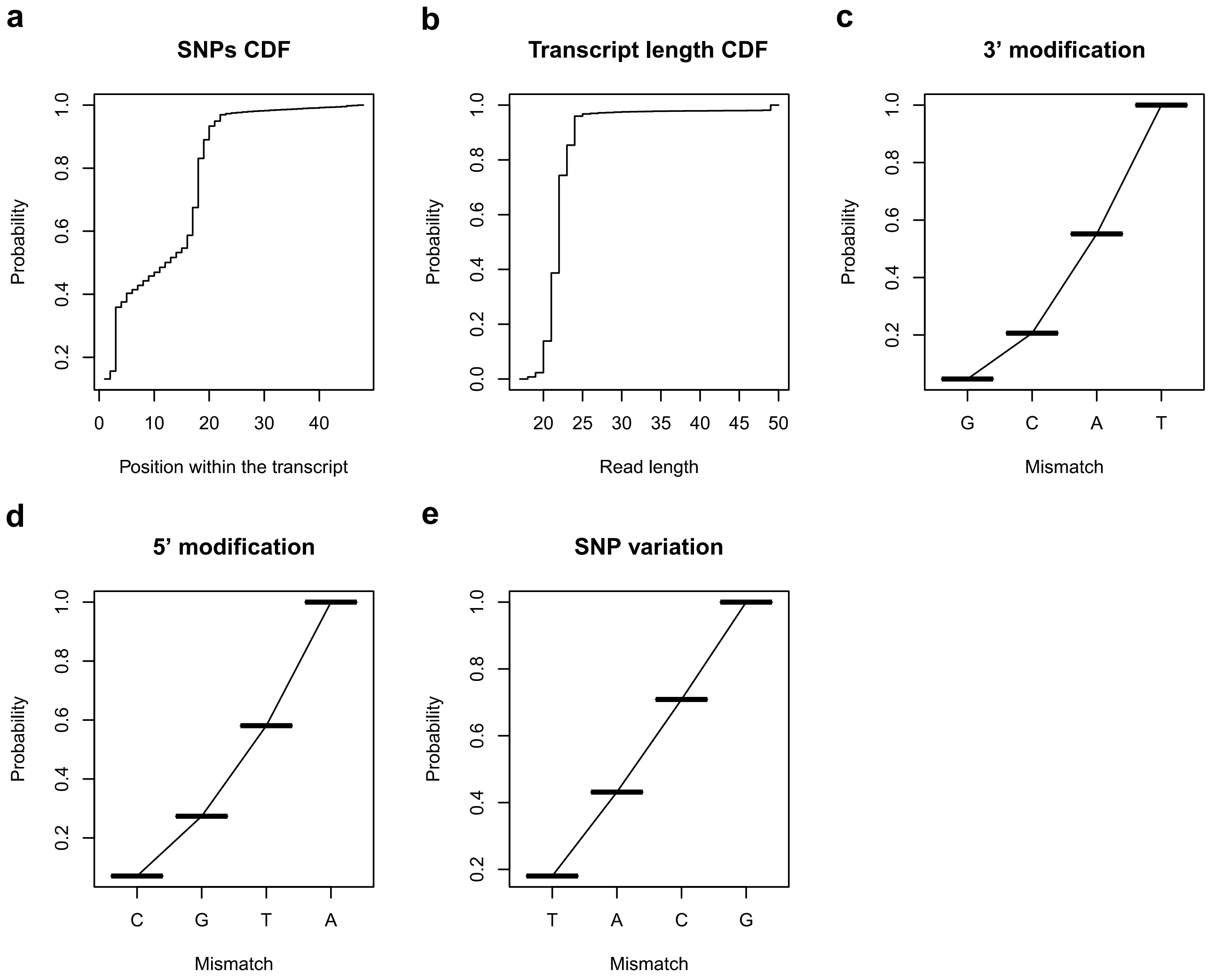

Supplementary Figure 5. CDFs of SNP positions, read lengths and probabilities of SNPs, non-template 3’ and 5’ additions, as estimated from the UARs present in the selected sRNA-Seq libraries. (a) Estimated CDF for the SNP position within the transcript area. (b) Estimated CDF for read length excluding miRNA lengths. Estimated probabilities for (c) 3’ or (d) 5’ non-template additions and for SNP nucleotide variation (e).

Supplementary Tables

Supplementary Table 1. sRNA-Seq datasets utilized in the multimaps analysis along with the related study and sample ID, and the relevant metadata information (cell/tissue type, sample type, and condition).

**Supplementary Table S1.** Real sRNA-Seq datasets utilized in the multimap analysis					
**#**	**Study ID**	**Sample ID**	**Cell/Tissue Type**	**Sample Type**	**Condition**
**1**	SRP049449	SRR1636959	hESC	Cells	Healthy
**2**	SRP049449	SRR1636960	hESC	Cells	Healthy
**3**	SRP060224	SRR2084358	MCF7	Cell-lines	Breast cancer
**4**	SRP060224	SRR2084359	MCF7	Cell-lines	Breast cancer, DHA-treated
**5**	SRP065616	SRR2889755	HepG2	Cell-lines	Liver cancer
**6**	SRP065616	SRR2889756	HepG2	Cell-lines	Liver cancer
**7**	SRP049590	SRR1642941	Liver	Tissue	Healthy
**8**	SRP049590	SRR1642943	Liver	Tissue	Healthy
**9**	SRP058632	SRR2038050	Liver	Tissue	Healthy
**10**	SRP058632	SRR2038051	Liver	Tissue	Healthy
**11**	SRP048606	SRR1596226	Liver	Tissue	Hepatoblastoma
**12**	SRP048606	SRR1596227	Liver	Tissue	Hepatoblastoma
**13**	SRP058632	SRR2038044	Heart	Tissue	Healthy
**14**	SRP058632	SRR2038045	Heart	Tissue	Healthy
**15**	SRP058632	SRR2038041	Brain	Tissue	Healthy
**16**	SRP058632	SRR2038042	Brain	Tissue	Healthy
**17**	SRP055439	SRR1813908	Colon	Tissue	Healthy
**18**	SRP055439	SRR1813909	Colon	Tissue	Healthy
**19**	ERP010082	ERR842903	Gallbladder	Tissue	Healthy
**20**	ERP010082	ERR842909	Gallbladder	Tissue	Healthy
**21**	SRP057590	SRR1988283	Lung	Tissue	Healthy
**22**	SRP057590	SRR1988284	Lung	Tissue	Healthy
**23**	SRP023531	SRR871609	Pancreas	Tissue	Healthy
**24**	SRP023531	SRR871671	Pancreas	Tissue	Healthy
**25**	SRP062601	SRR2174513	Skin	Tissue	Healthy
**26**	SRP062601	SRR2174514	Skin	Tissue	Healthy
**27**	ERP001908	ERR184035	Tongue	Tissue	Carcinoma
**28**	ERP001908	ERR184054	Tongue	Tissue	Carcinoma
**29**	SRP014020	SRR965791	Thyroid	Tissue	Cancer
**30**	SRP014020	SRR965792	Thyroid	Tissue	Cancer

Supplementary Table 2. sRNA-Seq datasets utilized in the simulation process along with the related study and sample ID, and the relevant metadata information (cell/tissue type, sample type, and condition).

**Supplementary Table S2.** sRNA-Seq datasets utilized in the simulation process.					
**#**	**Study ID**	**Sample ID**	**Cell/Tissue Type**	**Sample Type**	**Condition**
**1**	SRP059434	SRR2061810	Liver	Tissue	Healthy
**2**	ERR842903	ERR842903	Gallbladder	Tissue	Healthy
**3**	SRP006574	SRR191548	Breast	Tissue	Healthy

Supplementary Table 3. Transcripts excluded from the comparison analysis for real sRNA-Seq data. Seven transcripts with read count > 10,000 estimated by each examined tool were considered as outliers and removed from the comparison.

**Supplementary Table S5.** Transcripts excluded from the comparison analysis for real sRNA-Seq data						
**Transcript**	**HTSeq-Count**	**Manatee**	**miRDeep2**	**miRge**	**ShortStack**	**sRNAbench**
hsa-miR-26a-5p	12,826	13,020	12,961	13,004	12,968	12,980
hsa-miR-21-5p	76,203	229,881	228,759	229,645	229,014	229,372
hsa-miR-125a-5p	14,408	14,654	14,573	13,212	14,587	14,622
hsa-miR-99b-5p	43,811	47,224	46,969	43,200	47,015	47,109
hsa-miR-191-5p	32,305	34,601	34,351	33,359	34,394	34,494
hsa-miR-182-5p	20,083	26,152	26,010	20,188	26,060	26,044
hsa-miR-27b-3p	77,089	79,562	78,963	77,385	79,150	79,078

Supplementary References

1. Vlachos, I.S. et al. DIANA-mirExTra v2.0: Uncovering microRNAs and transcription factors with crucial roles in NGS expression data. *Nucleic acids research* **44**, W128-134 (2016).
2. Langmead, B., Trapnell, C., Pop, M. & Salzberg, S.L. Ultrafast and memory-efficient alignment of short DNA sequences to the human genome. *Genome biology* **10**, R25 (2009).
